# Beam hardening of *K*-edge contrast agents: a phantom study comparing clinical energy-integrating detector and photon-counting detector CT systems

**DOI:** 10.1186/s41747-024-00530-5

**Published:** 2025-03-19

**Authors:** Amir Pourmorteza, Arnaud Richard Choux, Thomas Wesley Holmes, U. Joseph Schoepf, Marly van Assen, Carlo De Cecco, Tilman Emrich, Akos Varga-Szemes

**Affiliations:** 1 Department of Radiology and Imaging Sciences, Emory University, Atlanta, GA USA; 2 Department of Biomedical Engineering, Georgia Institute of Technology, Emory University, Atlanta, GA USA; 3 Winship Cancer Institute, Emory University, Atlanta, GA USA; 4 Department of Radiology, Medical University of South Carolina, Clinical Science Building, Charleston, SC USA; 5 Department of Diagnostic and Interventional Radiology, University Medical Center of the Johannes Gutenberg-University, Mainz, Germany

**Keywords:** Artifacts, Bismuth, Gadolinium, Iodine, Tomography (x-ray computed)

## Abstract

**Background:**

Beam hardening (BH) artifacts negatively influence computed tomography (CT) measurements, especially when due to dense materials or materials with high effective atomic numbers. Photon-counting detectors (PCD) are more susceptible to BH due to equal weighting of photons regardless of their energies. The problem is further confounded by the use of contrast agents (CAs) with *K*-edge in the diagnostic CT energy range. We quantified the BH effect of different materials comparing energy-integrating detector (EID)-CT and PCD-CT.

**Methods:**

Pairs of test tubes were filled with dense CA (iodine-, gadolinium-, and bismuth-based) and placed inside a water phantom. The phantoms were scanned on EID- and PCD-CT systems, at all available tube voltages for the PCD scanner. Images were reconstructed with standard water BH correction but without any iodine/bone BH corrections. Virtual monoenergetic images (VMI) were calculated from PCD-CT data.

**Results:**

PCD-CT had higher CT numbers in all x-ray spectra for all CAs (*p* < 0.001) and produced larger cupping artifacts in all test cases (*p* < 0.001). Bismuth-based CA artifacts were 3- to 5-fold smaller than those of iodine- or gadolinium-based CA. PCD-CT-based VMI completely removed iodine BH artifacts. Iodine BH artifacts decreased with increasing tube voltage. However, gadolinium-based BH artifacts had a different trend increasing at 120 kVp.

**Conclusion:**

EID had fewer BH artifacts compared to PCD at x-ray tube voltages of 120 kVp and higher. The inherent spectral information of PCDs can be used to eliminate BH artifacts. Special care is needed to correct BH artifacts for gadolinium- and bismuth-based CAs.

**Relevance statement:**

With the increasing availability of clinical photon-counting CT systems offering the possibility of dual contrast imaging capabilities, addressing and comprehending the BH artifacts attributed to old and novel CT CAs grows in research and ultimately clinical relevance.

**Key Points:**

- EID-CT provides fewer BH artifacts compared to PCD-CT at x-ray tube voltages of 120 kVp and higher.
- *K*-edge CAs, such as those based on gadolinium, further confound BH artifacts.
- The inherent spectral information of photon counting detector CT can be used to effectively eliminate BH artifacts.

**Graphical Abstract:**

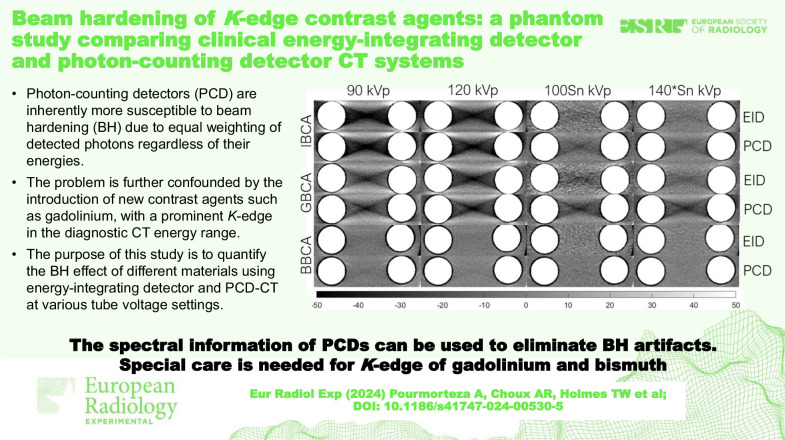

## Background

Beam hardening (BH) is a phenomenon that occurs when dense materials selectively absorb low-energy x-ray photons, altering the shape and mean energy of the x-ray beam [1]. This results in the formation of streaks, shadows, and cupping artifacts in the reconstructed computed tomography (CT) images. In cardiac CT, the presence of iodine-based contrast agents (IBCA) in the cardiac chambers and the descending aorta further exacerbates BH, potentially impacting the accuracy of myocardial CT measurements.

Photon-counting detectors (PCDs) are the recent technological advancements introduced in CT imaging. In cardiac CT, they offer improved spatial resolution, lower image noise, better image contrast, and spectral imaging capabilities [2–4]. However, they are particularly susceptible to BH due to their equal weighting of photons irrespective of their energies, as compared to energy-integrating detectors (EID), which weigh the incident photons by their energies [3, 5]. If left uncorrected, this inherent characteristic of PCDs can contribute to the amplification of BH artifacts in the reconstructed images.

Additionally, the introduction of novel CT contrast agents (CAs), such as gadolinium-based contrast agents (GBCAs), introduces an additional challenge in managing BH artifacts. GBCA exhibits a prominent *K*-edge within the diagnostic CT energy range, causing a significant alteration in the x-ray spectrum shape. This further complicates the behavior of BH artifacts and necessitates careful consideration of their mitigation strategies.

This study aimed to quantitatively assess the effects of BH induced by various CAs in both EID- and PCD-CT systems. By considering different tube voltages and beam prefiltration configurations, we aimed to gain insights into the influence of these parameters on the occurrence and severity of BH artifacts. Specifically, we will investigate the role of spectral information provided by PCDs in alleviating the BH artifacts.

## Methods

### Test objects

**Fig. 1:**
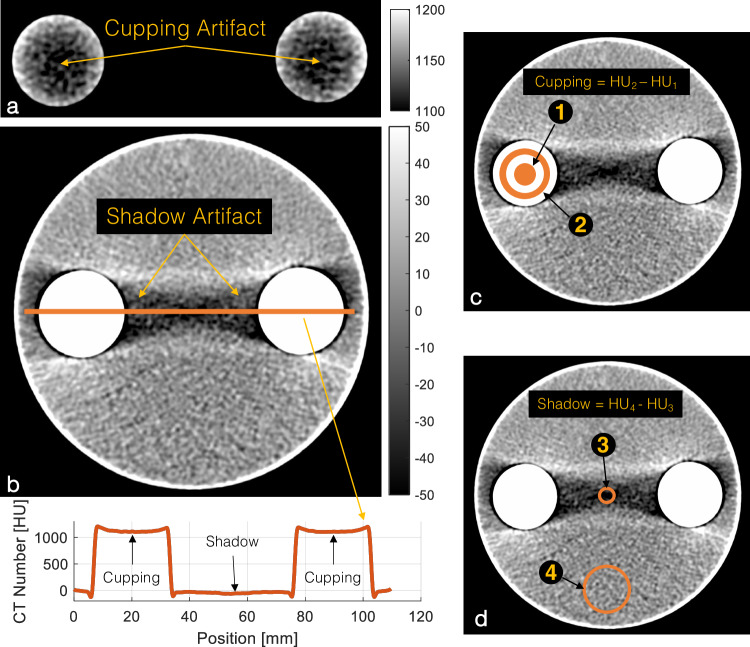
Sample 120-kVp EID image of the IBCA test object with iodine/bone BH correction intentionally turned off to accentuate the artifacts. **a** BH cupping artifact is visible as a decrease in the CT number of dense columns of IBCA. **b** BH shadow artifact presents a dark streak between the two IBCA inserts. The line profile shows both cupping and shadow artifacts. **c** Schematic of cupping artifact which is measured as the difference in average CT numbers of a circular ROI (#1) (diameter 0.75 cm) placed at the center of the test tube and a ring ROI (#2) (1.5 < diameter < 2.25 cm). **d** Shadow artifact quantified as the difference between the CT number of an ROI of 0.75 cm in diameter placed in the center of the shadow artifact (#3) and the CT number of background water (#4). CT, Computed tomography; IBCA, Iodine-based contrast agent; ROI, Region of interest

We prepared a series of phantoms made of two 50-mL conical polypropylene centrifuge tubes (ThermoFisher, Corning, New York, NY, USA) placed inside a 1,000-mL cylindrical polycarbonate jar (ThermoFisher, Corning) with a diameter of 11.2 cm such that the distance between their centerlines was 7 cm (Fig. 1). Both tubes were filled with one of the following four CAs: 1:10 dilution of iohexol as IBCA (Omnipaque 350, GE Healthcare, Chicago, IL), 1:5 dilution of gadabutrol as GBCA, (Gadavist, Bayer USA, Whippany, NJ, USA), and undiluted bismuth subsalicylate as bismuth-based contrast agent (BBCA) (Pepto-Bismol, Procter & Gamble, Cincinnati, OH, USA). The cylindrical jar was filled with deionized water.

### CT scanners and imaging parameters

The test objects were scanned on a first-generation clinical dual-source PCD-CT scanner (NAEOTOM Alpha, Siemens Healthineers, Forchheim, Germany), and a third-generation dual-source EID-CT scanner (SOMATOM Force, Siemens Healthineers, Forchheim, Germany) was used as a baseline. We acquired matched scans at 90 kVp, 120 kVp, and 100 kVp with Sn x-ray beam prefiltration (100Sn) for both systems. Additionally, we scanned the phantom at 140Sn for PCD and 150Sn for EID, as there were no other matching kVp settings available. This setting is referred to as 140*Sn in this study. Special attention was given to matching the scan and reconstruction parameters within the limitations of the two systems. All scans were acquired in helical mode (pitch 0.6) with 0.5-s gantry rotation time. The 90*Sn kVp, 120*Sn kVp, and 140*Sn kVp scans were dose-matched to a CT dose index volume (CTDIvol) of 15.7 mGy, and the 100Sn scans were matched to a CTDIvol of 6.8 mGy.

Energy thresholds were fixed at 25 keV and 65 keV for the PCD scans and images were created from all the detected photons above the lower threshold (threshold-1) used for comparison against EID. Additional virtual monoenergetic images (VMIs) and higher threshold (threshold-2) images were created using image domain energy bin information of PCD to assess improvements in BH. All EID and PCD scans were reconstructed using a weighted filtered-back projection algorithm with a soft quantitative convolution kernel (Qr40) and water BH correction. Iodine/bone BH correction was turned off to isolate the effects of BH on the images. Other imaging parameters included a slice thickness of 5 mm, a reconstruction diameter of 128 mm, and an image matrix size of 128 × 128, which resulted in an in-plane pixel spacing of 0.25 mm. EID images were reconstructed on the scanner (Syngo CT VA40, Siemens Healthineers, Forchheim, Germany) and the PCD images were created using an offline research reconstruction toolbox (ReconCT v13.8.4.0, Siemens Healthineers, Forchheim, Germany).

### Quantitative image analysis

Region-of-interest (ROI)-based measurements, as well as image and statistical analyses were performed using custom-made scripts in MATLAB (v2020a, Mathworks, Natick, MA, USA). Figure 1 shows the experimental setup and ROI locations. Each measurement was repeated on three different slices at least 1 cm apart from each other.

#### CT number measurements

Two circular ROIs of 2.25 cm in diameter were placed inside either of the test tubes containing the CAs (*n* = 2). A third ROI of 3 cm in diameter was placed away from the BH artifacts in the background water (Fig. 1d, ROI #4). The contrast-to-noise ratio (CNR) between water and each CA was calculated using the CA ROIs (*n* = 2) and the water ROI, with image noise defined as the standard deviation of the water ROI.

#### BH artifact metrics

We quantified two different manifestations of BH artifacts. “Cupping” artifacts are seen as decreased attenuation of areas surrounded by dense materials such as subcortical regions of the brain surrounded by the skull and inside vessels, and in this experiment, test tubes containing dense CAs (Fig. 1a). It was measured as the difference in average CT numbers of a circular ROI (diameter 0.75 cm) placed at the center of the test tube and a ring ROI (1.5 < diameter < 2.25 cm) as shown in Fig. 1c (ROI #1 and ROI #2). “Shadow” artifacts refer to the streaks and dark bands between two dense objects caused by BH. In this study, we quantified shadow artifacts as the difference between the CT number of a ROI of 0.75 cm in diameter placed in the center of the shadow artifact (Fig. 1d, ROI #3) and the CT number of background water (Fig. 1d, ROI #4), as explained in the CT number measurement section.

#### Simulated detected x-ray beam spectra

We simulated the shape of the detected x-ray spectra to better understand the effect of BH and *K*-edge of CAs on EID and PCD systems. We used the Beer–Lambert law to calculate the spectrum of a projection passing through 11 cm of water and 0.2-mm equivalent of ‘solid’ CA. These values do not necessarily match the phantom experiment. They were rather selected to provide a qualitative guide to explain the results of the phantom experiments. The EID spectra were multiplied by the energy of the detected photons, whereas the weighting factor for PCD was set at 1.

### Statistical analysis

Measurements are reported as mean ± standard deviation. Shapiro–Wilk test was used to assess the normality of distributions. Where appropriate, the Wilcoxon signed rank test or two-tailed paired *t*-test was used to compare measurements from the two scanners and between different tube voltage settings with *p* < 0.05 considered statistically significant.

## Results

**Fig. 2:**
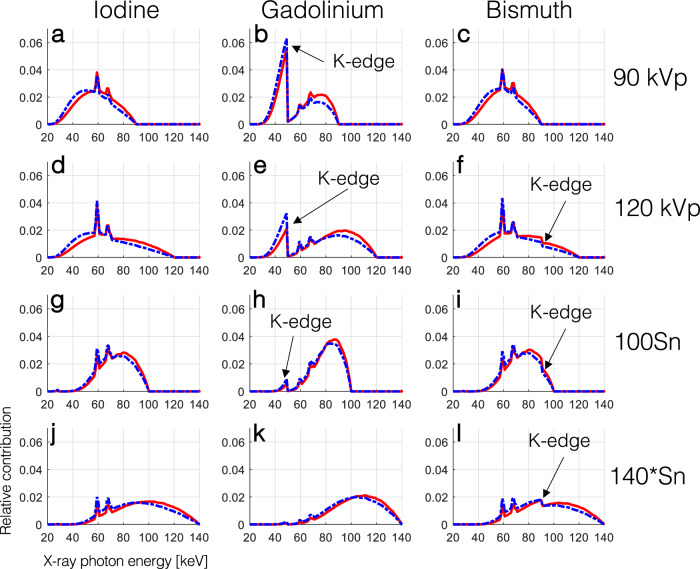
Simulated normalized detected x-ray spectra showing the relative contribution of photons of different energies to the signal detected by EIDs (solid red line) and PCD (dashed blue line) for a projection passing through 11 cm of water and 0.2-mm equivalent of ‘solid’ CAs. The effect of the *K*-edge of gadolinium and bismuth on the detected spectra is annotated with the black arrows

The effect of the *K*-edge of GBCA and BBCA on the detected spectra of EID and PCD systems is shown in Fig. 2. In all cases the EID spectra had more contribution in higher energies compared to the PCD spectra. While the *K*-edge of iodine (33.2 keV) is not visible in the spectra, that of gadolinium (50.2 keV) drastically alters the shape of the spectra, except in the 140*Sn setting. The *K*-edge of bismuth (90.5 keV) is also visible as a small dip in the spectra for all tube voltages larger than 90 kVp, although it did not drastically change their shape compared to those of iodine.

### CT number and CNR

**Fig. 3:**
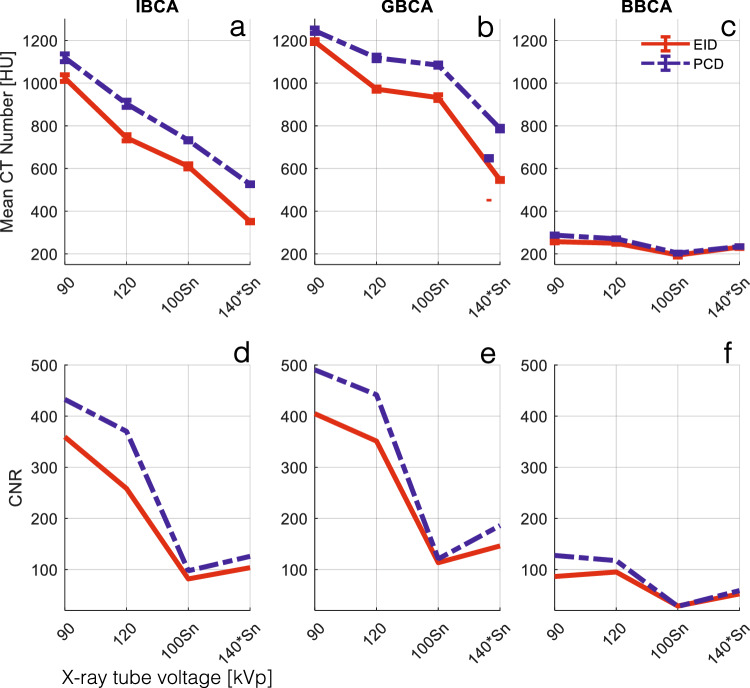
**a**–**c** Mean CT numbers of CAs used in this study with respect to different tube voltage and prefiltration settings for EID and PCD images. PCD CT numbers were significantly higher than those of EID for all tube settings and all CAs (*p* < 0.001). **d**–**f** CNR between water and the CAs in (**a**–**c**) IBCA, GBCA, and BBCA. PCD showed significantly higher CNR for all tube settings and all CAs (*p* < 0.004). BBCA, Bismuth-based contrast agent; CNR, Contrast-to-noise ratio; CT, Computed tomography; EID, Energy-integrating detector; GBCA, Gadolinium-based contrast agent; IBCA, Iodine-based contrast agent; PCD, Photon-counting detector

Figure 3a–c summarizes the CT numbers of CAs at different x-ray spectra. PCD-CT had significantly higher CT numbers in all x-ray spectra for all CAs (*p* < 0.001). This is attributed to the higher contribution of low-energy photons in the PCD spectra, as seen in Fig. 2. CNRs between water and CAs were also higher for PCD compared to EID in all cases.

### BH artifact metrics

**Fig. 4:**
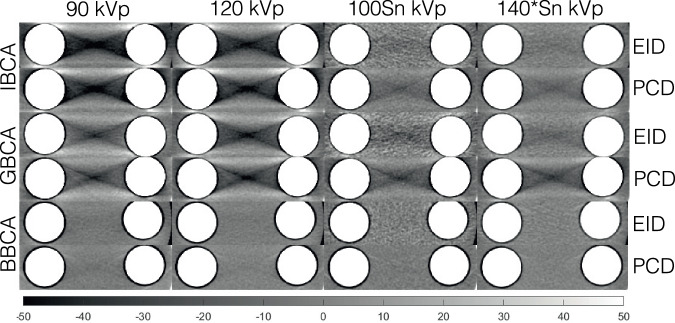
Cropped samples of all images used in this study showing the shadow BH artifacts for different CAs at different x-ray tube settings. Iodine/bone BH correction was intentionally turned off to accentuate the artifacts. BBCA, Bismuth-based contrast agent; EID, Energy-integrating detector; GBCA, Gadolinium-based contrast agent; IBCA, Iodine-based contrast agent; PCD, Photon-counting detector

**Fig. 5:**
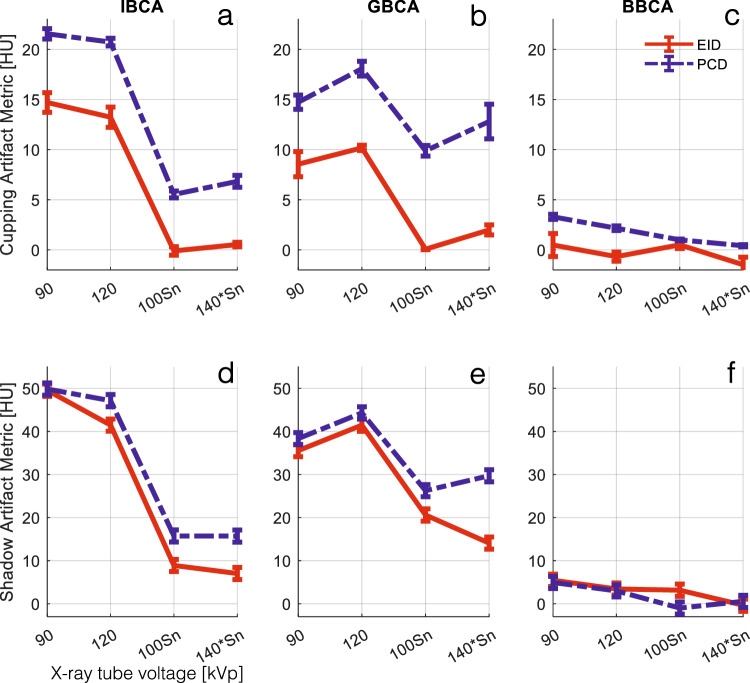
**a**–**c** Cupping BH artifact induced by the three CAs at different x-ray tube settings for EID and PCD images. Iodine/bone BH correction was intentionally turned off to accentuate the artifacts. The PCD images showed significantly higher artifacts across all CAs and tube settings (*p* < 0.004). **d**–**f** Shadow BH artifact induced by IBCA was significantly higher in PCD images for all tube settings except for 90 kVp, due to severe artifacts that saturated the CT number to -1,024 HU. Iodine/bone BH correction was intentionally turned off to accentuate the artifacts. Shadow artifacts induced by GBCA were significantly higher for PCD (*p* < 0.001). The magnitude of artifacts induced by BBCA was very small and not significantly different between the two detector systems, except at 100Sn (*p* < 0.008). BBCA, Bismuth-based contrast agent; CT, Computed tomography; EID, Energy-integrating detector; GBCA, Gadolinium-based contrast agent; IBCA, Iodine-based contrast agent; PCD, Photon-counting detector

Figure 4 depicts examples of the shadow artifact for all CA and x-ray spectra settings. Visual inspection shows more severe shadow artifacts in PCD images compared to all EID images. This is confirmed by quantitative analysis of the artifacts as reported in Fig. 5. PCD images produced significantly larger cupping artifacts in all test cases (*p* < 0.001). BBCA artifacts were 3- to 5-fold smaller than those of the IBCA and GBCA, and quantitative analysis was mostly dominated by image noise; therefore, for the rest of the study, we focused on IBCA and GBCA as they are more commonly used in the clinical use of CT. IBCA at 90 kVp produced the largest shadow artifact to the extent that it reached the lower end of CT numbers (-1,024 HU) in both EID and PCD images. GBCA BH artifacts were smaller than those of IBCA for 90- and 120-kVp settings. Sn prefiltration significantly decreased BH artifacts in IBCA and GBCA for both detector types.

### PCD spectrally derived images

**Fig. 6:**
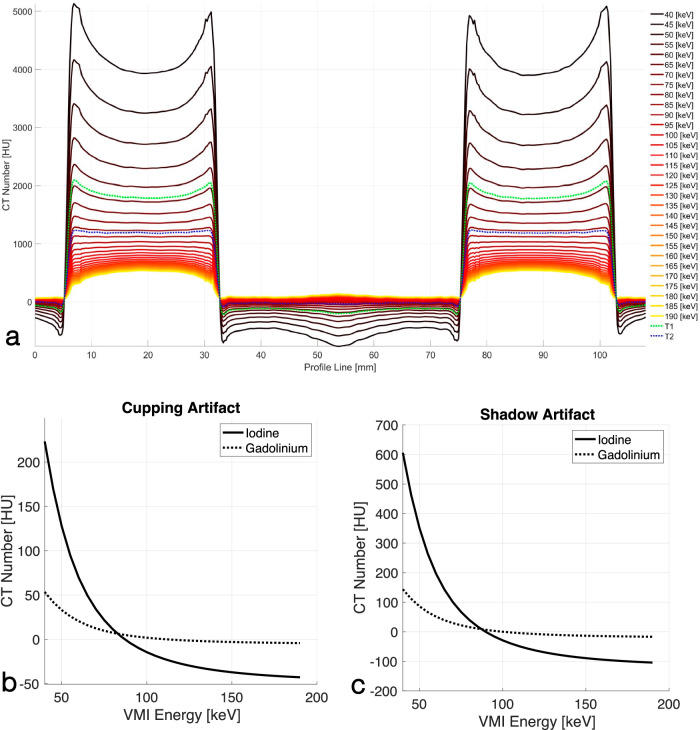
**a** Line profiles of PCD-derived VMIs and threshold-1 and threshold-2 images connecting the centers of two IBCA contrast columns showing cupping and shadow artifacts according to Fig. 1b. Iodine/bone BH correction was intentionally turned off to accentuate the artifacts. The cup shape flattens as the VMI energy increases. After a certain VMI energy, the flat shape is overcorrected into a cap. This is denoted by negative BH metrics in (**b**, **c**). Quantitative assessment of cupping (**b**) and shadow (**c**) artifacts with respect to VMI energy for IBCA and GBCA shows a decrease in the artifact with the increase of VMI energy, followed by overcorrection. CT, Computed tomography; EID, Energy-integrating detector; GBCA, Gadolinium-based contrast agent; IBCA, Iodine-based contrast agent; PCD, Photon-counting detector; VMI, Virtual monoenergetic image

The PCD acquisitions always carry spectral information embedded in the low-energy (threshold-1) and high-energy (threshold-2) thresholds. We generated threshold-2-images and VMIs in the 40–190-keV energy range at steps of 5 keV and evaluated their BH artifacts. Figure 6a shows line profiles of the spectrally derived PCD images for the IBCA phantom drawn according to Fig. 1b. It qualitatively shows that there are VMIs that remove both cupping and shadow BH artifacts. This is quantitatively shown in Fig. 6b, c. The cupping artifact for IBCA reaches zero at an energy between 85 keV and 90 keV for IBCA and at an energy between 110 keV and 115 keV for GBCA. Similarly, the shadow artifacts were eliminated at an energy between 85 keV and 90 keV for IBCA, and between 100 keV and 105 keV for GBCA. Furthermore, while the BH artifacts for IBCA were overcorrected in VMIs above the said energy levels, indicated as negative BH metrics, we did not observe such a strong trend for GBCA. Threshold-2-images also showed a significant decrease in BH metrics (*p* < 0.001) compared to threshold-1-images.

## Discussion

In this study, we showed that in PCD-CT the inherent equal weighting of all detected x-ray photons, regardless of their energy, leads to improved contrast and CNR in images created from all detected photons (threshold-1) compared to conventional EID-CT images as seen in Fig. 3. As shown in Fig. 2, the same phenomenon leads to higher BH artifacts in PCD-CT compared to EID-CT, regardless of the CA used if iodine/bone BH correction algorithms are not used. This was evaluated in Figs. 4 and 5. Next, we showed that by taking advantage of the embedded spectral information in PCD acquisitions and generating VMIs, it is possible to eliminate the BH artifacts in the absence of iodine/bone BH correction algorithms. Intuitively, VMI generation can be thought of as changing the weighting factor of the detected photons, based on their energies such that BH artifacts are removed.

It is also possible to generate VMIs from dual-energy EID scans [6]. Studies have reported comparable performance in VMIs and iodine density estimation between EID and PCD systems [7, 8], and therefore we did not repeat them here. Furthermore, when using EID scanners, selecting the dual-energy mode prior to scanning often involves a compromise concerning temporal resolution, which is clinically relevant, especially in cardiovascular imaging. In contrast, PCD-CT always provides spectral information, which can be accessed and utilized if needed once the CT examination is completed. This study of a commercial PCD-CT scanner confirms previous findings investigating the BH performance of a prototype PCD-CT scanner [9] and simulation studies [10] for artifacts caused by IBCAs.

VMIs are commonly generated using two-material decomposition algorithms [11]. They offer the potential for standardization and enhanced quantitative imaging when using EIDs and PCDs [8, 12, 13]. However, the presence of *K*-edge CAs such as GBCA can complicate this process if appropriate PCD energy threshold settings and multi-material decomposition algorithms capable of capturing the *K*-edge characteristics of the CA are not utilized [14]. Similarly, *K*-edge-specific algorithms may be required to correct BH artifacts caused by *K*-edge CAs.

This study examined the distinct behavior of BH artifacts induced by IBCA and GBCA. While it is widely acknowledged that higher x-ray tube voltages alleviate BH artifacts caused by bone and iodine, our findings showed a reversal of this trend in the presence of GBCA, as demonstrated in Fig. 5. Interestingly, lower tube voltages resulted in reduced BH artifacts in both EID and PCD images. Moreover, despite our GBCA sample having a higher CT number compared to the IBCA sample at 90 kVp, the BH artifacts were significantly lower for GBCA, regardless of the detector technology employed.

The application of Sn prefiltration resulted in a reduction of BH artifacts in both systems for IBCA and GBCA. However, it is worth noting that the filtration of the *K*-edge of gadolinium by Sn (as depicted in Fig. 3) only led to a modest decrease in the BH artifacts of GBCA at the 140*Sn tube voltage.

GBCA is the agent of choice for magnetic resonance imaging, and without *K*-edge imaging its use in CT has been limited to special cases in CT for patients allergic to iodine or those with compromised renal function. Furthermore, it requires doses up to five times larger than those used for magnetic resonance imaging [15]. Adverse effects of GBCA have been largely attributed to linear compounds with the list significantly lowered by macrocyclic GBCAs [16]. Nevertheless, reducing the dose of CAs is a worthy endeavor from patient safety and cost-saving points of view. GBCA seems to be the only Food and Drug Administration-cleared CA that could be used in dual-contrast imaging tasks alongside IBCAs.

*K*-edge imaging with PCD-CT has shown promise to improve the CNR of GBCA and significantly reduce the dose required for CT imaging [14]. Aside from these concerns, GBCA might be the CA of choice for many CT applications. It has been used along with IBCA in dual-contrast imaging tasks that resolve two different perfusion phases of an organ, *e.g*., arterial and delayed myocardial enhancement of the heart [17], the liver [18–20], and the kidneys [21], in one scan, thereby reducing radiation dose and providing co-registered maps of different perfusion phases of the same organ. It has also shown promise in detecting endoleaks [22, 23] and as a standalone CA in cardiovascular imaging [24]. Tuning the energy thresholds of PCDs to the *K*-edge of gadolinium improves its CNR and simplifies material decomposition in tasks such as CA *versus* calcium separation necessary in atherosclerotic plaque characterizations. This is due to the better spectral separation of gadolinium and calcium compared to that of iodine in the energy ranges of diagnostic CT. BBCAs, especially in the form of bismuth subsalicylate, could potentially have off-label use as an oral CA, along with intravenous IBCA [25] or GBCA, for detecting endoleaks and gastrointestinal bleeds in abdominal PCD-CT [21, 26].

Therefore, *K*-edge imaging can be impactful in a wide range of applications from basic science and animal research to routine clinical imaging tasks. As the cost of clinical PCD-CT scanners continues to drop to levels comparable to high-end CT scanners before the advent of PCD-CT, and with over 100 scanners now installed in clinical centers, the barriers to their clinical adoption are being significantly reduced. Additionally, these scanners are fully reverse compatible with EID-CT protocols, further easing the transition to PCD-CT.

This study has several limitations. Firstly, we did not employ iodine and bone BH correction algorithms intentionally to amplify the artifacts and investigate their behavior in the presence of *K*-edge materials. It is anticipated that the use of such algorithms would greatly diminish the artifacts. Next, we used a geometrically simple phantom to showcase and accentuate BH artifacts for accurate measurements. While the physics and effects of BH remain the same *in vivo* compared to our study, further studies with anthropomorphic phantoms may be warranted.

Additionally, the impact of x-ray photon scatter caused by IBCA and GBCA was not explored and merits separate analysis [27, 28]. Moreover, while GBCAs are utilized in research PCD-CT studies, including dual- and multi-contrast imaging [17, 19, 21, 23], their adoption in clinical practice is growing. Traditionally GBCAs are used in CT of patients with contraindications to IBCA [29]. However, *K*-edge imaging of GBCAs may lead to a new and more efficient application of traditional and nanoparticle-based GBCAs [30]. It should also be noted that while this experiment was performed on a CdTe PCD-CT scanner, the same concepts and arguments hold true for all PCDs including CdZnTe [30, 31] and deep-silicon [32–35] detectors as long as image-domain spectral processing is performed. Projection-domain spectral processing may mitigate BH artifacts even further. Lastly, the maximum available tube voltage was 150 kVp *versus* 140 kVp for EID *versus* PCD systems, respectively. This created a slight difference in the x-ray spectra at this setting, however as shown in Figs. 4–6, the trend of higher BH artifacts in PCD remained the same compared to the previous voltage settings.

In summary, PCDs are more vulnerable to BH artifacts compared to EIDs. However, the advantage of PCD scans lies in their inherent spectral information, which can be utilized to effectively mitigate BH artifacts even in the absence of BH correction algorithms. Additionally, when studying *K*-edge CAs, it is crucial to consider the nonlinear effect of BH in relation to tube voltage and consider the implementation of dedicated *K*-edge material decomposition and BH correction algorithms as necessary.

## Data Availability

The datasets generated and/or analyzed during the current study are not publicly available due to active and ongoing research and grant writing by the authors on this subject. However, they are available from the corresponding author upon reasonable request.

## Abbreviations

BBCA: Bismuth-based contrast agent
BH: Beam hardening
CA: Contrast agent
CNR: Contrast-to-noise ratio
CT: Computed tomography
CTDIvol: CT dose index volume
EID: Energy-integrating detector
GBCA: Gadolinium-based contrast agent
IBCA: Iodine-based contrast agent
PCD: Photon-counting detector
ROI: Region of interest
VMIs: Virtual monoenergetic images
